# Vitamin D, allergies and asthma: focus on pediatric patients

**DOI:** 10.1186/1939-4551-7-27

**Published:** 2014-12-10

**Authors:** Auro Della Giustina, Massimo Landi, Federica Bellini, Mariangela Bosoni, Giuliana Ferrante, Marzia Onorari, Alessandro Travaglini, Giuseppe Pingitore, Giovanni Passalacqua, Salvatore Tripodi

**Affiliations:** Department of Pediatrics, National Healthcare System Fornovo, Parma, Italy; Department of Pediatrics, National Healthcare System ASL TO1, Turin, Italy; Pediatric Unit Department of Gynecologic, Obstetric and Pediatric Sciences, University of Bologna, Bologna, Italy; Pediatric Unit, G. Fornaroli Hospital, Magenta, Milan, Italy; Department of Sciences for Health Promotion and mother and child, University of Palermo, Palermo, Italy; Department of Prato, Environmental Protection Agency of Tuscany (ARPAT), Prato, Italy; Aerobiological Monitoring Center, University of Tor Vergata, Rome, Italy; Allergology Unit, G.B.Grassi Hospital, Rome, Italy; Allergy and Respiratory Diseases, IRCCS San Martino-Ist-University of Genoa, Pad. Maragliano, L.go R Benzi 10, 16133 Genoa, Italy; Department of Pediatrics and Allergy Unit Sandro Pertini Hospital, Rome, Italy

**Keywords:** Vitamin D, Allergic diseases, Immunomodulation, Supplementation, Asthma, Rhinitis, Pediatric allergy

## Abstract

In recent years, the interest of the scientific world towards vitamin D gradually increased, and several studies have been conducted to dissect its possible role in modulating the development/course of allergic diseases. Also, Vitamin D supplementation has been assessed as a beneficial approach for treating allergies in some, but not all studies. We reviewed herein the available and relevant literature concerning the possible links between Vitamin D, its supplementation and allergic diseases. A literature search was made independently by the Authors, identifying articles for a narrative review. As per literature, Vitamin D plays a key role in calcium and phosphate metabolism, and it is essential for bone health in infants, children and adolescents. However, there is presently insufficient evidence to support vitamin D supplementation for prevention or treatment of allergic diseases in infants, children and adolescents, concerning allergic rhinitis, asthma, food allergy and atopic dermatitis.

## General background

**Figure 1:**
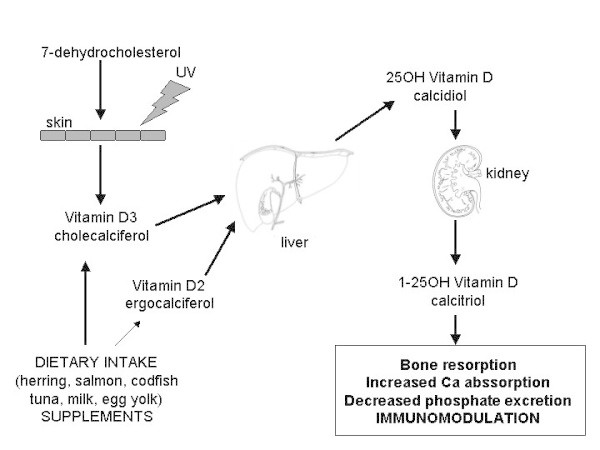
**General metabolism of vitamin D, precursors and derivatives.**

During the two last decades, the scientific interest on the Vitamin D system progressively increased. Apart from the well-known role of this vitamin in bone and calcium metabolism, recent observations have suggested its possible role as a pivotal immune-modulator also in allergic diseases, including asthma [1], and this aspect could assume a particular relevance in pediatric patients. A growing body of literature underlined that vitamin D plays an important role in the general function/regulation of immune system, expecially concerning lymphocyte function, T cell antigen receptor signaling and activation, cytokine production [2–4]. Based on these observations, the vitamin has been suggested as a potential factor affecting incidence, severity and course of asthma and allergic diseases [5, 6], thus envisaging also preventive roles. It is also true that some studies suggested that high serum levels of vitamin D may increase the risk of allergic disorders [7–12]. Cholecalciferol, and its metabolites, are more properly hormones that can be synthesized by the human body (Figure 1). Ultraviolet radiations determine the photochemical conversion in the skin of 7-dehydrocholesterol into cholecalciferol (Vitamin D3). Subsequently, in the liver, mitochondrial and microsomal enzymes similar to cytochrome P450 determine its hydroxylation in position 25 to get 25-hydroxy-cholecalcipherol (calcidiol), that is usually named and assayed as Vitamin D (VD) since it represents the most abundant circulating form, with a long half-life. Approximately, 88% of VD circulates bound to specific binding proteins, or bound to albumins, whereas only 0,03% is free. The second hydroxylation, that is necessary for having an active hormone, occurs in kidney, where VD is converted in the active form (1–25 hydroxyVD, calcitriol) [13].

Up to recent times, it was argued that the conversion of VD into its active metabolite could exclusively occur in the kidney. Latest discoveries have brought to light how other cells in different organs express receptors for vitamin D. Typical examples are represented by T and B lymphocytes, monocytes, antigen presenting cells (APC) including macrophages and dendritic cells [14]. In the matter of this, it is established that vitamin D exerts its effects on the immune system, especially increasing the expression of cathelicidins hCAP18, important defense factor against pathogens of the respiratory tract [15]. Cathelicidins produced by neutrophils and epithelia, after a signal mediated by inflammatory cytokines, would seem to determine the chemotaxis of the cells of innate immunity by activating an inflammatory response against several microorganisms. Moreover, vitamin D may stimulate the production of cationic peptides, beta-defensin 2 and 4 [15].

The supposed antiallergic effects of VD may in part be ascribable to the action on dendritic cells, favoring the production of IL-10 and reducing the production of IL-12 [16]. A serum level of VD ≥ 50 nmol/L is considered sufficient, values < 50 nmol/L insufficient, and < 40 nmol/L possibly at risk for disease. To ensure an adequate intake of vitamin D, the American Academy of Pediatry has raised the daily recommended intake for children and adolescents [17], to a dose of 400 IU up to 12 months of age and 400–600 over 12 months [18], recommending that this supplementation should begin during the first days of life.

Concerning allergic diseases, the available studies provided conflicting results. Certainly, in addition to serum levels of VD, other factors may play a crucial role in the development of allergies and asthma, including environment and genetics. In this regard, an interesting aspect concerns the latitude [19]: high latitudes (evaluated in consideration of residence at time of birth and interview), which are characterized by lower ultraviolet irradiation, may be associated with a lower frequency of allergy, while a higher ultraviolet exposure (lower latitudes), was associated with an increased likelihood of having a history of allergic rhinitis or asthma or both conditions during childhood. Of course, these represent only a cross-sectional study, and do not provide a direct evidence that sun-exposure is related to VD and allergy.

Interventional studies with VD in patients with immune-mediated diseases were not fully exhaustive. The extent of involvement of vitamin VD-dependent and VD-independent pathways in homeostasis and regulation of immune system in diseases still needs to be explored [20].

### Vitamin D and allergic rhinitis

There are few studies related to allergic rhinitis. An Australian study [21] after observing that the prevalence of allergies increases in percentage, with the decrease of latitude, tried to describe each latitudinal variation in the prevalence of allergy in childhood and to assess, in parallel, the possible association between ultraviolet irradiation and VD, in relation with allergic rhinitis, asthma, or both conditions. The conclusions have shown that the inverse association between latitude and asthma is not dependent on ultraviolet but attributable to other climatic factors such as temperature. It was also reported that supplementation with cod liver oil before 15 years of age is associated with an increased likelihood of having asthma and allergic rhinitis. Another study performed in Norway displayed a direct association between VD deficiency and male sex for the development of allergic rhinitis, whereas females resulted protected according to VD levels [22]. The research provides a rationale for the evaluation of the possible central role of early vitamin D supplementation for the development of allergy in childhood. Different patterns of sun exposure are probably also involved in allergic sensitization.

### Vitamin D, asthma and wheezing

Concerning the relationship between VD and wheezing, a prenatal VD deficiency seems to predispose to wheezing as well as to subsequent asthma, adversely affecting the development of the lung as well as of the fetal immune system [11]. An adequate intake of VD during pregnancy would seem to exert a protective action on the onset of childhood wheezing and asthma, especially in the male offspring [23]. This appears to be due to the synergistic action of VD and 17-beta-estradiol, with a final reduction of the catabolism of VD [24]. Children born from mothers with VD deficiency during pregnancy are predisposed to an increased risk of recurrent wheezing at 3 years of age. The additional intake of 100 IU of vitamin D in the first and second trimester of pregnancy is associated with a lower risk of asthma and wheezing during childhood [9]. The possible influence of genotype-associated VD binding proteins, makes the link between asthma and VD even more complex [25].

Asthma, in its allergic phenotype, is classically determined by an increased activity of TH2 cells resulting in the production of IgE and inflammatory cytokines causing airway hyperresponsiveness with a predominantly eosinophilic inflammation. In recent years, many studies focused the possible protective role of vitamin D against bronchial asthma [26]. Nonetheless in a Spanish birth cohort, higher maternal VD at 12 weeks’ gestation was not associated with wheezing at 1 year or 4 years or asthma at age 4 to 6 years [7]. Also, a recent study from UK found no association with dietary vitamin D and wheeze, asthma, or sensitization [8]. Using either maternal or cord blood as biomarker for fetal VD exposure, an inverse associations with the risk of developing respiratory and allergic disease was found [9–12] .

Gupta et al. showed an inverse relationship between serum concentration of VD and severity of asthma attacks, number of exacerbations and consumption of inhaled corticosteroids (ICS); the same study also showed how optimal levels of VD were associated with a good control of the disease [27]. Searing et al. observed that children with asthma had overall insufficient serum levels of vitamin D, with an inverse correlation between VD, total IgE and skin prick tests (SPT) positivity, and a direct association with increased corticosteroid usage [28]. From a functional point of view, it has also been shown that a deficiency state involves the reduction of the forced expiratory volume in the 1st second (FEV1) in patients with mild to moderate asthma.

VD would also modulate various cytokines induced effects through different cells of the immune system with a dose-dependent action. Reasonable doses of VD inhibit the production of both TH1 and TH2 cytokines, while high concentrations seem to even amplify the TH2 responses [29]. Moreover, VD, in association with glucocorticoids, may increase directly or indirectly the production of anti-inflammatory cytokines such as IL 10 [30]. Concerning the airway remodeling in asthma, some studies showed that VD can affect remodeling through a direct effect on the proliferation of smooth muscle cells of the airways, also influencing their growth and contractility [31].

### Vitamin D and steroid resistance

The molecular mechanisms of glucocorticoid resistance in children are probably different, unclear and not yet clearly defined. A congenital steroid resistance, resulting from any genetic mutation of the receptors is rare [32]. On the contrary, acquired resistance is more common, and often can be overcome by increasing the dose, this increasing also the risk of side effects. There are several potential mechanisms underlying resistance to steroid therapy that have been mainly studied in adult subjects [33, 34]. The pathophysiological mechanism underlying allergic responses involves the initial participation of the innate immunity APC resulting in the activation of the TH2 lymphocytes response. The regulatory T cells, through the production of cytokines such as IL-10 and TGF-beta, negatively modulate the activation of this immune response that contribute to airways inflammation and hyperreactivity. A functional reduction of the regulatory T lymphocytes activity has been associated with the resistance to glucocorticoid therapy as well [35].

More recently, several Authors underlined that VD may be involved in the increase of regulatory T lymphocytes recruitment [36]. In conditions of VD deficiency regulatory T-lymphocytes were reduced not only in number but in overall functionality and effectiveness. A small pilot study has also suggested that the intake of VD in people with asthma would increase the response to therapy with dexamethasone [36]. We could than assume that vitamin D may potentially increase the therapeutic response to glucocorticoids in those subjects that exhibit resistance to steroids.

Some mechanisms (Table 1) have been proposed to explain how VD can interact with steroid therapy. Sutherland et al. [37] showed an association between low levels of vitamin D and impaired lung function, increase of airway hyperresponsiveness and reduction of glucocorticoid response in a group of patients with moderate to severe asthma. VD can also modulate at genomic level the transcription of genes for proteins with inflammatory activity [38–40]. Another mechanism potentially involved in steroid resistance is the ability of VD to adjust the expression of genes involved in inflammatory phenomena, in relation to the regulation of glucocorticoid receptors themselves [41].

**Table 1:** **Potential mechanisms of interaction between vitamin D and GC**

-	Enhanced dexamethasone (DEX)-induced expression of IL- 10 by T regulatory cells
-	Preincubation of T cells with both IL-10 and vitamin D3 overcame defects in DEX-induced CD41 T cell IL-10 production
-	Possible mechanism in which vitamin D3 reversed ligand-induced down-regulation of the GC receptor
-	High concentrations of vitamin D were instead put in relation with the increased expression of MPK-1 protein by blood mononuclear cells

In vitro, physiological concentrations of VD added to dexamethasone significantly increase the expression of proteins MPK-1 in peripheral blood mononuclear cells compared to dexamethasone alone, suggesting that the addition of vitamin D could reduce the effective dose of dexamethasone required. Hence, it is important to emphasize that a treatment with vitamin D in an asthmatic patient may not only lead to a significant improvement in clinical symptoms, but also to a tapering of the steroid dose thus avoiding the well-known side effects [38].

### Vitamin D and asthma exacerbations

It is well known that viral infections of the respiratory tract lead to an increase of asthma exacerbations both in children and in adults [42]. Rhinovirus infections, for example, induce an inflammatory state in the airways, not only increasing the severity of asthma exacerbation, but also leading to infections running definitely with a greater severity than in non asthmatic patients [42].

Emerging new evidences brought to light as subjects with inadequate intake of vitamin D exhibit a higher number of respiratory infections per year and that these may onset with greater severity [43].

One prospective cohort study measured the different concentrations of VD in 198 adult subjects observing how individuals with VD concentrations less than 38 ng/mL had a risk of viral infections of the respiratory tract doubly increased [44]. The EDEN [45] cohort study found no significant association between cord-blood VD levels and the risk of developing asthma. Also, it was evidenced that a VD deficiency status make more prone to exacerbations of asthma children than adults [45–47]. According to these data it is clear how important is an early identification of vitamin D deficiency status, and also a prompt setting of adequate supplementation, to prevent several diseases both in adults and in children.

### Vitamin D and food allergy

Although the extra-skeletal role of VD is definitely intriguing and should not be underestimated, at this moment there is a lack of consistent data facing the issue of VD in the development/prevention of food allergies. One recent cross-sectional study [48] on more than 500 infants with proven food allergy showed a direct relation with VD deficiency and disease, although more detailed immunological parameters (e.g. food-specific immunoglobulins) were not investigated. Another study evidenced that high vitamin D levels in pregnancy and at birth may contribute to a higher risk for food allergy [49], this standing against VD supplementation to protect against allergy. Another birth cohort study on 650 infants could not detect an epidemiological relation between VD deficiency and risk of food allergy, although an association with specific genetic assets was seen [50]. Considering the large amount of literature regarding the mechanisms associated with atopic diseases, an evaluation of serum levels of VD and finally its supplementation must be regarded as a further opportunity to understand and treat atopic diseases, but well-designed studies on VD supplementation to prevent food allergies are needed [51].

### Vitamin D and atopic dermatitis

Some studies on this subject indicated an inverse relationship between the prevalence and/or severity of atopic dermatitis and VD levels. Furthermore, studies have shown that, in individuals with dermatitis and VD deficiency, a supplementation reduces the severity of the disease [52, 53]. However, data are not consistent, as this correlation has been found, but only in patients with allergic sensitization [54, 55], and other studies found no or an inverse correlation between VD deficiency and atopic eczema [56, 57]. In addition, a recent controlled trial found no visible effect of VD supplementation on clinical severity of the disease [58]. Of note, cathelicidin, an antimicrobial innate protein, is presently regarded as a possible biomarker linking VD and innate immune regulation in atopic dermatitis [59, 60].

### Vitamin D and anaphylaxis

It would seem to exist an interesting relationship between latitude and episodes of anaphylaxis as reported in the records of the emergency rooms and the sales of self-injectable epinephrine. In his study Mullins showed that both parameters were higher in the southern regions of Australia [61]. Nonetheless, a clear and direct relationship within VD and anaphylaxis has been so far demonstrated or explored in detail.

### Concluding remarks

Taken together the available literature, it is not yet possible to confirm or refute the direct role of VD in the development/worsening of allergic diseases in pediatric age and in newborns, neither to assign a relevant role to VD use in an immunological therapy setting. Many confounding and still unidentified variables are present in the various studies. There is an overwhelming experimental evidence that vitamin D acts on the function of immune cells, but the complexity of this system, cannot be applied to the general population, and no specific nutritional guideline can be issued in the setting of allergy. Also, it is not yet possible to recommend an absolute strategy for the use of VD in the therapy of asthma and allergic diseases, or in prevention [62–65]. Clinical trials and population-based prospective studies are needed, in order to better understand the molecular mechanism by which VD may affect immunological disorders and their development. [3]. VD plays a key role in calcium and phosphate metabolism and is essential for bone health in infants, children and adolescents; however, up to now there is insufficient evidence to support vitamin D supplementation to obtain other benefits [58]. In conclusion, pediatricians should pay closer attention to vitamin D levels in allergic children and their parents for a better disease management.
